# Micro-scale H_2_–CO_2_ Dynamics in a Hydrogenotrophic Methanogenic Membrane Reactor

**DOI:** 10.3389/fmicb.2016.01276

**Published:** 2016-08-17

**Authors:** Emilio Garcia-Robledo, Lars D. M. Ottosen, Niels V. Voigt, M. W. Kofoed, Niels P. Revsbech

**Affiliations:** ^1^Section of Microbiology, Department of Bioscience, Aarhus UniversityAarhus, Denmark; ^2^Biological and Chemical Engineering, Department of Engineering, Aarhus UniversityAarhus, Denmark; ^3^Danish Technological InstituteAarhus, Denmark

**Keywords:** hydrogen, microsensor, methane, biogas, membrane reactor, methanogenesis, pH, CO_2_

## Abstract

Biogas production is a key factor in a sustainable energy supply. It is possible to get biogas with very high methane content if the biogas reactors are supplied with exogenous hydrogen, and one of the technologies for supplying hydrogen is through gas permeable membranes. In this study the activity and stratification of hydrogen consumption above such a membrane was investigated by use of microsensors for hydrogen and pH. A hydrogenotrophic methanogenic community that was able to consume the hydrogen flux within 0.5 mm of the membrane with specific rates of up to 30 m^3^ H_2_ m^-3^ day^-1^ developed within 3 days in fresh manure and was already established at time zero when analyzing slurry from a biogas plant. The hydrogen consumption was dependent on a simultaneous carbon dioxide supply and was inhibited when carbon dioxide depletion elevated the pH to 9.2. The activity was only partially restored when the carbon dioxide supply was resumed. Bioreactors supplied with hydrogen gas should thus be carefully monitored and either have the hydrogen supply disrupted or be supplemented with carbon dioxide when the pH rises to values about 9.

## Introduction

The increasing concern about the rise in atmospheric CO_2_ is driving intense research into alternative energy sources. The biological production of methane in bioreactors reduces the organic waste from several agricultural or industrial facilities and the product is a gas that may be used as fuel in combustion engines, for power generation or transport, and in the longer term as a fossil free raw material for chemical synthesis. However, the biogas produced in an anaerobic reactor is typically composed of only 50–70% of CH_4_, 30–50% CO_2_, and minor amounts of other gasses (Ghorbanian et al., 2014). The high CO_2_ concentration reduces the quality of the raw biogas and makes it unsuitable for transport purposes. To reduce the amount of CO_2_ in the produced gas and at the same time increase the CH_4_ yield, an injection of H_2_ into the reactor has been proposed (Luo and Angelidaki, 2012). The injection of H_2_ would provide the electron donor needed to reduce more CO_2_ to CH_4_ by the hydrogenotrophic methanogenic community. This could upgrade biogas to a quality approaching natural gas (Luo and Angelidaki, 2013; Wang et al., 2013; Bensmann et al., 2014; Ghorbanian et al., 2014).

Renewable energy sources such as wind or sunlight are subject to weather conditions. As the supply from renewable sources and the demand fluctuates, the market price of electricity changes hourly at the established power trading platforms, and in periods with much wind the prices may be very low or even negative^1^. One method to efficiently utilize renewable energy in periods with low cost is to produce H_2_ by electrolysis (Sherif et al., 2005). However, the direct use of H_2_ as fuel or in fuel cells has several disadvantages due to complex transportation and storage (Luo et al., 2012). Hydrogen gas may, however, be used for production of methane by microbiological or chemical means (Stempien et al., 2015), as CH_4_ has higher volumetric energy content and the infrastructure is already available for its use. The use of H_2_, produced by water electrolysis, for conversion to CH_4_ enables the indirect accumulation of wind or solar power as a storable gas. When the conversion is done by microbes in a biogas plant it furthermore utilizes a readily available source of biologically formed CO_2_. Chemical conversion would, on the other hand, require a concentrated source of CO_2_ obtained from for instance combustion of biomass, fossil fuels, or cement production.

Injection of H_2_ into a working biogas reactor does not necessarily imply substantial structural modifications and the essential microbial community of hydrogenotrophic methanogens is generally well developed in biogas reactors (Demirel and Scherer, 2008). It could therefore be expected that an implementation of H_2_ addition to biogas reactors should be straightforward. However, a number of experiments in small scale reactors have revealed several lacks in our knowledge about the complex interaction of microbial, physical, and chemical processes occurring in the reactors, resulting in an inefficient conversion of the injected H_2_. One critical limitation of the process is a low solubility of H_2_, resulting in a low rate of gas/liquid transfer by bubbling (Luo et al., 2012; Bensmann et al., 2014; Ghorbanian et al., 2014). The use of permeable membranes has been proven to be more efficient, resulting in the full consumption of the injected H_2_ and a higher CH_4_ content of the produced biogas (Wang et al., 2013; Díaz et al., 2015). Another limiting factor is that the consumption of CO_2_ due to methanogenesis may cause an increase in pH to values higher than 9, resulting in the destabilization of the whole reactor (Luo et al., 2012; Xu et al., 2014; Hu et al., 2015). Addition of acidic substrates to the reactor to balance the pH has therefore been applied to maintain high CH_4_ production rates (Luo and Angelidaki, 2013; Wang et al., 2013; Ghorbanian et al., 2014). An alternative would be to reduce the H_2_ input to levels not resulting in almost complete CO_2_ depletion and associated high pH.

The gas permeable membrane reactor has been shown to be an efficient design for the bio-methanization of H_2_ (Wang et al., 2013; Díaz et al., 2015). The microbial transformation is expected to occur in a biofilm growing on the membrane, being supplied with H_2_ by diffusion through the membrane. By use of microsensors it is possible to measure relevant chemical parameters such as H_2_, H_2_S, and CH_4_ in such biofilms (Revsbech, 2005) with high temporal and spatial resolution. The interference of H_2_S on the signal from electrochemical H_2_-sensors could have prevented a microscale study of H_2_ transformations in this kind of biofilm, but recently a H_2_ microsensor with a H_2_S trap has been developed (Nielsen et al., 2015).

The aim of this study was to investigate how hydrogenotrophic methanogenesis is regulated at a microscale when H_2_ is supplied to biogas reactors. We studied an anaerobic membrane reactor where H_2_ was supplied through a gas permeable membrane, but the results may also illustrate the microenvironment and metabolic rates around H_2_ bubbles if H_2_ is supplied by bubbling. Hydrogen and pH microprofiles were measured in the microbial community overlying a silicone membrane and the spatial microdistribution of processes was calculated from the profiles. The time course development of microprofiles and activity was investigated in both fresh cattle manure at 20°C and in biomass from a mesophilic biogas reactor at 38°C. The effects of various rates of CO_2_ supply on the microdistribution of H_2_ and pH and on the rate of H_2_ consumption were examined in detail.

## Materials and Methods

### Experimental Set-up: Bioreactor and Microsensor Measurements

**FIGURE 1:**
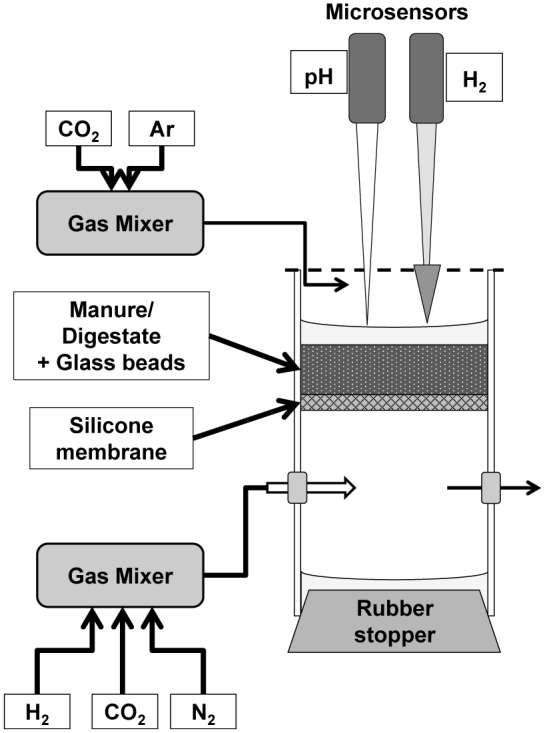
**Silicone membrane anaerobic bioreactor design**.

A small scale silicone membrane reactor similar to the one described by Revsbech (1989) was used to provide a controllable environment in terms of H_2_ supply (**Figure 1**). This design deviates substantially from the silicone-tubing membrane reactors used by Wang et al. (2013) and Díaz et al. (2015) as it was decided to exploit a geometry that was possible to analyze with microsensors and allowed reliable mathematical analysis. The reactor consisted of two cylindrical chambers (3 and 6 cm length) consisting of Plexiglas (i.d. 4 cm) separated by a silicone rubber membrane. The membrane was made by coating a stainless steel mesh (mesh size 1 mm) with silicone rubber (Silastic RT adhesive 732, Down coring, Midland, MI, USA), resulting in a total thickness of 1.5 mm. The membrane provided a stable base for the substrate while simultaneously being highly permeable to gasses (Robb, 1968). The chamber below the membrane was used as a reservoir of gasses diffusing up into the upper compartment. The bottom was closed by a rubber stopper and two holes in the sides (8 mm), closed with rubber stoppers, allowed the continuous flushing with a humidified gas flow of 100 mL/min through hypodermic needles of the chamber with a gas mixture controlled by a gas mixer (Brooks Instruments D.V., Holland). To ensure anaerobic conditions in the upper chamber, the upper opening, except for a small opening for microsensor insertion, was closed with Parafilm while it was continuously flushed with humidified Argon or an Ar:CO_2_ mixture (∼50–100 mL/min). Maintaining a higher density of the flushing gas than the surrounding air was essential to avoid turbulent mixing of atmospheric air into the chamber. The anoxic conditions were confirmed by an oxygen microsensor with the tip placed in the upper headspace.

A recently improved H_2_ microsensor (Nielsen et al., 2015) was used to measure the distribution of H_2_ diffusing from the silicone membrane. The new sensor has no interference from H_2_S, which can be present at relatively high concentrations (up to mM range) in both raw and digested cattle manure. The size of the tip (70–100 μm) allowed the measurement of profiles with a high spatial resolution of 100 μm. The relatively long diffusion path from the exterior to the internal anode results in a negligible effect of variations in turbulence and diffusive properties in the analyzed medium. The H_2_ sensors applied thus exhibited a signal difference between vigorously stirred and stagnant medium of <3% but a response time of about 30 s for 90%. pH microprofiles were also measured in some of the experiments. The pH microsensors had a pH-sensitive tip length of 150 μm, allowing for a similar vertical resolution as the H_2_ measurements. Hydrogen gas and pH were measured simultaneously in some of the experiments (see below) by the use of one sensor of each kind, measuring in the same reactor. The H_2_ microsensor was connected to a pico-ammeter (PA2000, Unisense) whereas the pH microelectrode was connected to a custom-built mV-meter (Aarhus University) and to an external Ag/AgCl reference electrode positioned inside the chamber. Microsensors were mounted together in a computer-controlled micromanipulator (MC-232, Unisense) while the signals were recorded through an A/D converter (ADC-216, Unisense) using the Sensor TracePro software (Unisense).

Profiles of H_2_ were modeled, using the numerical procedure described by Berg et al. (1998). The numerical fitting of the measured H_2_ generate profiles with the spatial distribution of the H_2_ consumption rates, being expressed as nmol cm^-3^ s^-1^. The obtained data were also depth-integrated to get the total rates being expressed as μmol cm^-2^ h^-1^. The diffusion coefficient in water was taken from the diffusion coefficient tables compiled by Unisense^2^. The solubility of H_2_ in water was calculated (Crozier and Yamamoto, 1974) to be 778 and 728 μmol L^-1^ at 22 and 38°C, respectively. The effective diffusion coefficient in the sieved fresh manure was assumed to be equal to the one in water. The effective diffusion coefficient in the glass beads biofilm was calculated according to Revsbech (1989) being 0.63 times the value in the water.

### Development of an Active H_2_ Consuming Community in Fresh Cattle Manure

The initial activity and development of an active H_2_ consuming community was followed in fresh cow manure at room temperature (22°C). Cow manure was collected in a dairy cattle farm near Aarhus, Denmark, brought to the laboratory and sieved applying a mesh-size of 2 mm, to remove large particles that could damage the sensor. The manure was then added to the bioreactor described previously to get a layer of about 15 mm on top of the silicone membrane, ensuring the complete anoxic conditions at the bottom. The bottom chamber of the reactor was flushed with H_2_ (100%) while the upper chamber was flushed with Ar. The system was left to equilibrate for 2 h after which H_2_ microprofiles were measured every 5 h until a steady state was obtained as judged from several consecutive profiles.

The evolution of the H_2_ consumption rates showed the same pattern as bacterial growth and therefore, the rates were fitted to the Gompertz equation modified by Zwietering et al. (1990), adapted to the measured rates:

$$Ln(\frac{R}{R_{o}})=R_{m}\times \exp\left[-\exp\left[((\mu_{m}\times e)/A)\times (\lambda -t)+1\right]\right]$$

Where: *R* is the H_2_ consumption rate, Ro is the initial rate, Rm is the maximum rate, μ_m_ is the maximum specific growth rate (h^-1^), λ is the lag time (h) and *A* is the asymptote, being equal to Ln(Rm/Ro). The model was fitted to the experimental data by minimum least squares fitting, using the Solver command in Excel (Kemmer and Keller, 2010).

### Activity and CO_2_ Dependence of H_2_ Consumption Rates in the Digested Content of a Biogas Reactor

The development of an active H_2_ consuming sludge matrix and the dependence on rates of CO_2_ supply were investigated in sludge from a mesophilic anaerobic biogas reactor (hereafter referred as digestate) that was sieved through a 2 mm screen and mixed with glass beads (40–60 μm diameter, 3 M to provide a stable matrix for profile analysis. The digested manure is a liquid and turbulent mixing due to convection would make the results difficult to interpret. The mixture of digestate and glass beads was added to the bioreactor (the bottom chamber being flushed with 100% H_2_) and the glass beads were allowed to settle down for 10 min. The supernatant was then removed with a pipette to form a 4–5 mm active layer above the membrane and the upper chamber closed with Parafilm. The thickness of the layer was reduced in order to reduce the capacity for CO_2_ production of the system and then be more dependent of the external gasses manipulations. The bioreactor was then immersed in a temperature-controlled water bath at 38°C. A water saturated gas flow controlled by a gas mixer enabled changes in gas composition in both upper and lower chambers.

Two experiments with different types of gas supply manipulations were carried out:

#### Development of the Community and Time Course after CO_2_ Depletion

To follow the development of an active H_2_ consuming community in the digestate, the bottom chamber was supplied with 100% H_2_ while the upper one was supplied with a mixture of Ar:CO_2_ (70:30%) to avoid CO_2_ limitation. After 1 h of equilibration, H_2_ microprofiles were measured every 2 h. After 20 h, the CO_2_ supply was terminated while keeping the same measuring frequency for another 20 h.

#### Dependence of H_2_ Consumption of CO_2_ Supply

The bottom compartment was supplied with a mixture of H_2_:N_2_ (71:29%) while the upper one was supplied with 100% Ar during the whole experiment. After 1 h for equilibration, H_2_ and pH microprofiles were measured hourly. Once stable conditions were reached (by measuring two consecutive profiles with apparently identical H_2_ distributions), the CO_2_ proportion in the gas supply of the bottom chamber was consecutively increased to the following values: 3, 6, 12, 14, 21, and 29% (reducing the N_2_ but keeping constant the H_2_ percentage). Finally, the flushing was returned to the initial H_2_:N_2_ mix and the system was followed until steady-state profiles were approached.

## Results

### Development of an Active H_2_ Consuming Community in Cattle Manure

**FIGURE 2:**
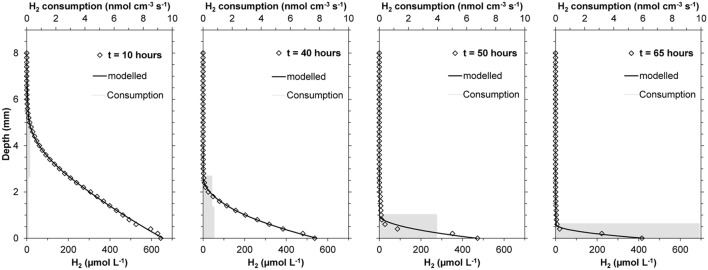
**Evolution of H_2_ profiles during incubation of fresh cow manure on top of a silicone membrane at 22°C, showing the evolution of the real data (♢), modeled profiles (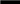), and estimated volumetric H_2_ consumption rates (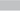)**.

**FIGURE 3:**
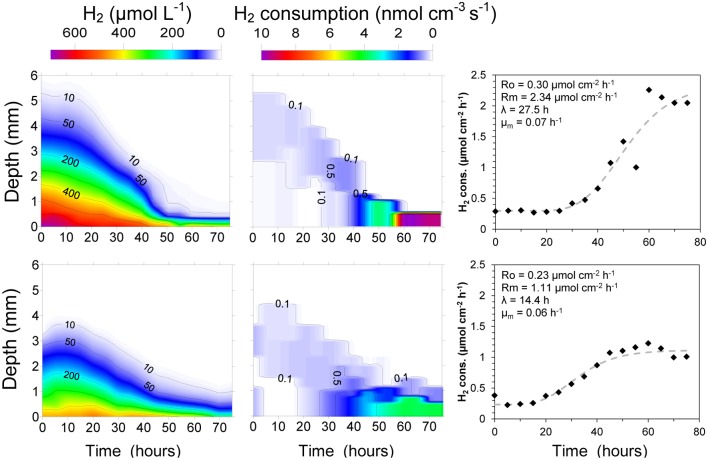
**Evolution of integrated H_2_ consumption rates in two samples of fresh cow manure incubated on top of a silicone membrane at 22°C.** The increase in consumption rates were interpreted as growth of the methanogenic population with the parameters of the Gompertz equation shown above the simulated rise in H_2_ consumption rates.

An initially large H_2_ penetration in the cattle manure suggested relatively low activity of the microbial community already present in the manure (**Figures 2** and **3**). H_2_ microprofiles were similar during the first 15–25 h in both experiments conducted (**Figure 3**) showing H_2_ diffusion from the silicone membrane into the first 3–4 mm of the manure. During this period, H_2_ consumption rates were low (<0.25 nmol cm^-3^ s^-1^, equivalent to 0.55 m^3^ H_2_ m^-3^ manure d^-1^) and extended along the 3–4 mm where H_2_ could be measured. The integrated H_2_ consumption rate was 0.22–0.30 μmol cm^-2^ h^-1^ (1.4–1.8 L m^-2^ d^-1^) (**Figure 3**) during this initial period. After this lag period, lasting for 14.4 to 27.5 h according to the Gompertz model, the H_2_ profiles changed quickly, resulting in a progressive decrease in H_2_ penetration and increase in rates of consumption (**Figures 2** and **3**). The H_2_ consumption was finally concentrated in a thin layer of less than 0.5 mm, with maximum rates of 5.7–9.5 nmol cm^-3^ s^-1^ (corresponding to 12.5–20.9 m^3^ H_2_ m^-3^ manure d^-1^). Assuming that the H_2_ consumption rates were proportional to the populations density of methanogens it can be calculated from the Gompertz equation that the maximum specific growth rates were 0.06–0.07 h^-1^. After 60 h, integrated consumption rates reached stable values of 1.11–2.05 μmol cm^-2^ h^-1^, corresponding to 6.8–12.5 L m^-2^ d^-1^ (**Figures 2** and **3**).

### Activity and CO_2_ Dependence of H_2_ Consumption Rates in the Slurry of a Biogas Reactor

**FIGURE 4:**
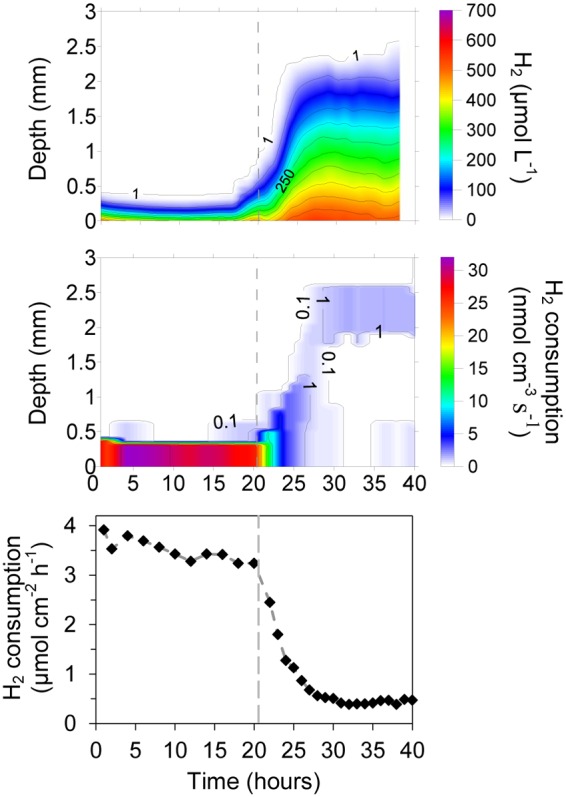
**Development of H_2_ consumption in a digestate/glass bead matrix placed on top of a silicone membrane at 38°C.** Below the membrane the gas consisted of humidified H_2_ gas, whereas an Ar:CO_2_ (70:30%) mix was applied in the headspace above the matrix. After 20 h the CO_2_ supply was interrupted, resulting in a 90% reduction in the H_2_ consumption of the matrix.

The development of an active H_2_ consuming community was followed using digestate from a mesophilic anaerobic reactor. Contrary to the experiments with fresh cattle manure, the H_2_ profiles showed a high rate of consumption already at the time of the first profile measurement 1 h after the set-up of the system (**Figure 4**). The H_2_ diffusing out of the membrane was quickly consumed in a layer thinner than 0.5 mm with rates varying from 24.5 to 31.9 nmol cm^-3^ s^-1^ (53.9–70.2 m^3^ H_2_ m^-3^ digestate d^-1^). The integrated rates were also relatively constant with values of 3.24–3.92 μmol cm^-2^ h^-1^ (19.8–24.0 L m^-2^ d^-1^) (**Figure 4**).

**FIGURE 5:**
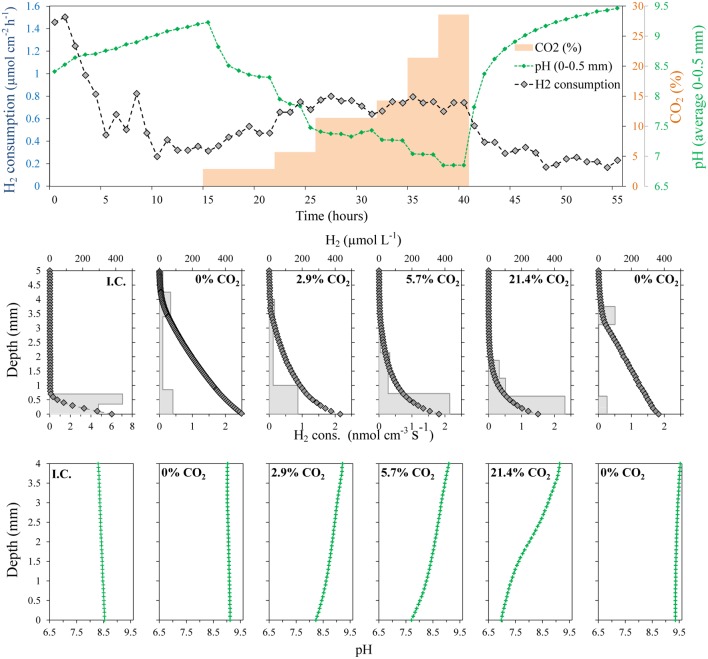
**Evolution of H_2_ concentration profiles, integrated H_2_ consumption rates and pH in a ∼ 4 mm thick methanogenic matrix located above a gas permeable membrane.** The matrix was supplied with H_2_ by diffusion from a reservoir below the membrane having a H_2_ partial pressure of 0.71 bars, while the CO_2_ partial pressure was varied. The temperature was 38°C. No CO_2_ was supplied at the initial conditions (I.C.).

After 20 h of continuous H_2_ supply thought the silicone membrane, and CO_2_ supply from the upper headspace (Ar:CO_2_ 70:30%), the CO_2_ supply was stopped, resulting in a rapid decrease in H_2_ consumption rates of the microbial community (**Figure 4**). The penetration of H_2_ into the matrix thus increased from a layer thinner than 0.5 mm to more than 2.5 mm. After 6–7 h, the H_2_ consumption seemed to be localized in the upper part of the matrix with maximum rates around 1.6 nmol cm^-3^ s^-1^ (3.5 m^3^ H_2_ m^-3^ digestate d^-1^). The integrated rates were also stable, showing low consumption values of 0.39–0.47 μmol cm^-2^ h^-1^ (2.4–2.9 L m^-2^ d^-1^) (**Figure 4**). These rates may, however, be overestimates as the consumption determined from the change in profile slope in the upper part of the matrix may be an artifact due to measurements in a meniscus at the transition from liquid to gas (**Figures 4** and **5**).

Due to the clear effect of CO_2_ supply on the activity of the digestate in terms of H_2_ consumption, the effect of the CO_2_ concentration on the measured rates was also investigated (**Figure 5**). To get dependence from the external CO_2_ supply instead of the internal CO_2_ stock, the system was initially supplied with a gas mixture of 71% H_2_:29% N_2_ to deplete the inorganic carbon in the system. Similar to the previous experiment with CO_2_ starvation (**Figure 4**), the H_2_ consumption rates decreased progressively, reaching relative stable and low values around 0.35 μmol cm^-2^ h^-1^ (2.1 L m^-2^ d^-1^) after 5–6 h (**Figure 5**). The CO_2_ starvation also resulted in substantial changes in the pH values throughout all depths. The pH thus increased progressively from about 8.5 to 9.1 throughout the digestate (**Figure 5**).

**FIGURE 6:**
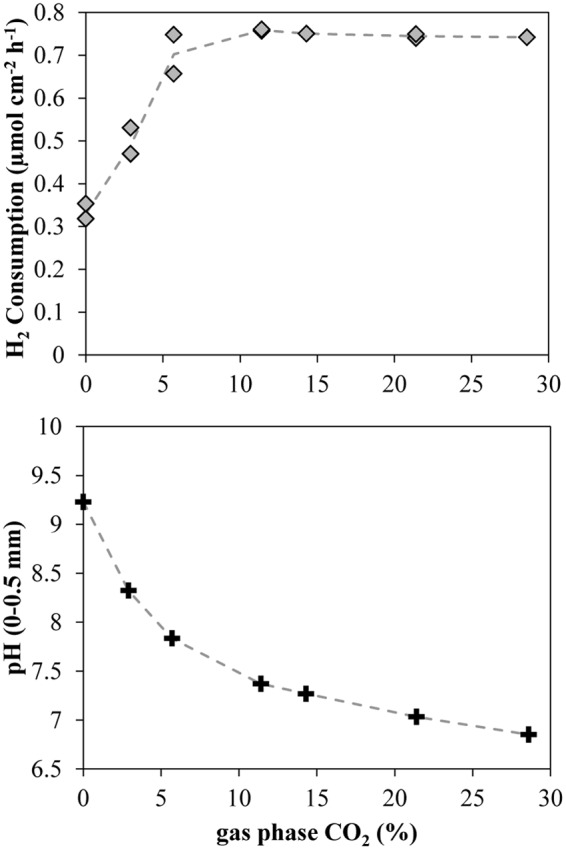
**Integrated H_2_ consumption rates and close-to-membrane pH in a 38°C methanogenic matrix exposed to various CO_2_ concentrations.** Lines connect the average of the data points.

A progressive increase in CO_2_ concentration in the gas supplied to the chamber below the silicone membrane (keeping H_2_ at 71%) resulted in a progressive increase in the H_2_ consumption rates. After a period of several hours without CO_2_, an increase of CO_2_ to 2.9% produced a clear increase in the H_2_ consumption rates, reaching a stable value of 0.5 μmol cm^-2^ h^-1^ (3.1 L m^-2^ d^-1^) after 5 h (**Figure 5**). The next increase in CO_2_ % to values of 5.7% also produced a clear increase in the H_2_ consumption rates to a value of 0.75 μmol cm^-2^ h^-1^ (4.6 L m^-2^ d^-1^). However, further increases did not produce any clear increase in H_2_ consumption rates and a slight decrease could even be observed at the highest concentrations (**Figures 5** and **6**). The spatial distribution of the H_2_ consumption was also modified. The H_2_ consumption at the initial conditions (**Figure 5**) was mainly localized in a narrow layer of ca. 0.5 mm with a high activity of 5.9 nmol cm^-3^ s^-1^ (13.0 m^3^ m^-3^ d^-1^). However, the depletion and subsequent resumed CO_2_ supply resulted in lower rates (maximum of 2.63 nmol cm^-3^ s^-1^ at 21.4% of CO_2_) distributed in a broader area of 2–3 mm (**Figure 5**).

An increase of the CO_2_ supply produced a substantial pH decrease in the layers closest to the membrane (**Figures 5** and **6**). Just 2.9% CO_2_ thus reduced pH close to the membrane from a value about 9.2 during CO_2_ depletion to 8.2. At 5.7% CO_2_ the value further decreased to 7.7 and at 21.4% it reached a pH of 7. The pH in the upper parts of the matrix stayed at a relatively high level during the increase in CO_2_ supply from below, due to the flushing of the headspace with pure argon.

## Discussion

### Vertical Micro-structure of a Hydrogenotrophic Biofilm (Conceptual Model)

**FIGURE 7:**
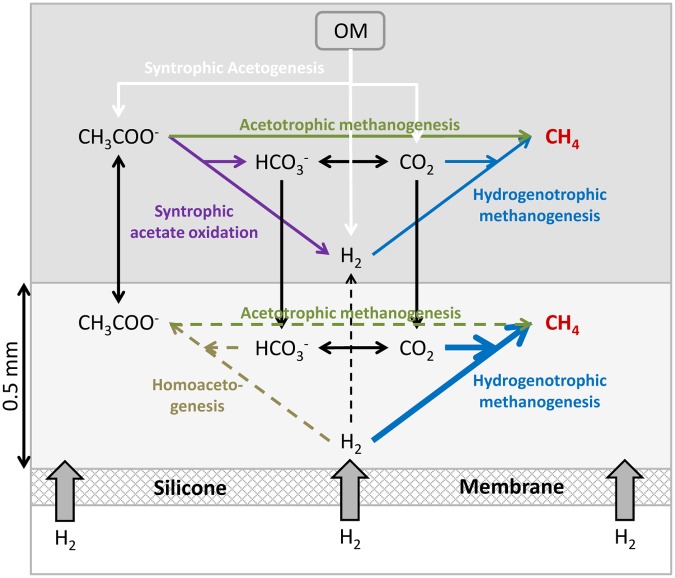
**Conceptual model of the microbial processes occurring in a membrane reactor for the supply of H_2_ to a methanogenic biofilm.** The light gray layer of 0.5 mm illustrates a region (“biofilm”) supplied with H_2_ by diffusion from the silicone membrane. OM: Organic matter.

In a standard methanogenic reactor, the decomposition of the various organic compounds results in acetogenesis with acetate (CH_3_COO^-^) and CO_2_+H_2_ being the final products of fermentation processes. These compounds are then converted to methane by the acetoclastic and hydrogenotrophic methanogenesis, respectively (**Figure 7**). Hydrogenotrophic methanogeneis is highly energetically favorable and the concentration of H_2_ is usually maintained at very low values allowing for the syntrophic acetogenesis (Schink, 1988). However, the use of acetate is less energetically favorable and acetate is thus generally accumulating to higher concentrations. At low H_2_ concentrations, the syntrophic acetate oxidation can also occur, converting acetate to CO_2_+H_2_, which then can be transformed into methane.

The localized supply of H_2_ into an anaerobic reactor through a membrane is to a major extent modifying the balances and reactions taking place in the nearby microbial community. The high concentrations of H_2_ close to the membrane inhibit syntrophic acetogenesis and syntrophic acetate oxidation, as these processes are no longer energetically favorable (Demirel and Scherer, 2008). Homoacetogenesis producing acetate from H_2_ and CO_2_ and hydrogenotrophic methanogenesis should then be dominant processes and consume all the H_2_ supplied through the membrane. From **Figures 2–5** it appears that complete depletion may occur over a distance as small as 0.5 mm. Among these two processes, the hydrogenotrophic methanogenesis is more energetically favorable and it has been shown to be the dominant process in reactors feed with H_2_, outcompeting homoacetogenesis (Díaz et al., 2015). The H_2_ consumption processes both use CO_2_ resulting in an increase of pH (**Figure 5**), which could lead to inhibition of the processes.

The processes of syntrophic acetogenesis and homoacetogenetic oxidation can take place in the bulk reactor outside the zone of H_2_ supply from the membrane. Both processes produce CO_2_ and H_2_ which can feed the CH_4_ production by hydrogenotrophic methanogens. Acetogenesis and homoacetogenesis in the bulk reactor create good conditions for acetoclastic methanogenesis (Demirel and Scherer, 2008), producing CO_2_ and thus contributing to the CO_2_ supply of the high hydrogenotrophic activity near the membrane.

The structure and control of the activity near the membrane is thus regulated by the supply of H_2_ and CO_2_. The H_2_ supply to the zone must be balanced with the CO_2_ supply from local production and diffusion from nearby areas. An excess of H_2_ supply as compared to the supply of CO_2_ leads to the increase in pH which results in the inhibition of several microbial processes and an instability of the system (Wang et al., 2013; Bensmann et al., 2014). A control of H_2_ supply and the maintenance of moderate pH values around 8 have been demonstrated to mediate an almost complete conversion of CO_2_ to CH_4_ in membrane reactors (98–99% CH_4_ in the produced biogas) with stable microbial communities, without affecting the microbial degradation of organic matter (Wang et al., 2013), inferring that most of the reactor was still characterized by very low H_2_ concentrations allowing for syntrophic acetogenesis (Wang et al., 2013). However, an additional external CO_2_ supply could allow for higher amounts of methane being produced.

### Development of Hydrogenotrophic Community at Low Temperature

The use of small-scale biogas reactors is an increasing practice in livestock farms from developing countries (Pham et al., 2014). The digestion of animal manure at relatively low temperatures (25°C) has been proven to be enough for a modest biogas production (<1 m^3^ biogas/Kg volatile solids). However, the efficiency of the process is relatively low and start-up times longer than 1 month are needed (Zeeman et al., 1988; Alkaya et al., 2010; Pham et al., 2014). The injection of H_2_ to untreated wet manure is, however, a different scenario. The methanogenic community is already present in the manure (Zeeman et al., 1988; Demirel and Scherer, 2008) and an injection of H_2_ will boost the growth of the community. In our small reactor, we measured a H_2_ consumption rate of 20 m^3^ H_2_ m^-3^ manure day^-1^ (**Figure 3**) after 3 days. The H_2_ consumption may, however, not result in a 100% conversion to CH_4_. A large fraction (up to 70%) of the H_2_ consumption has been reported to be used for growth of the methanogens and not for methane production during the start-up phase of a reactor (Díaz et al., 2015). The only about 60 h needed to obtain maximum activity in the raw manure (**Figure 2**) is a very short time period as compared to the start-up times needed for cattle manure reactors (Zeeman et al., 1988). Considering the integrated consumption rates as a proxy for the size of the microbial community, maximum values of 0.05–0.08 h^-1^, corresponding to doubling times of 9–14 h of the hydrogenotrophic microbial community, were measured (**Figure 3**). These values are in the range of the optimal doubling times of several species of mesophilic methanogens (Demirel and Scherer, 2008). Our data illustrate that the growth rate of the methanogenic community in raw cattle manure is high and the long start up times for methanogenic digestors fed with cattle manure thus cannot be due to the methanogens.

The zone with measurable H_2_ concentrations compared to the total thickness of the applied matrix (about 1.5 cm) was relatively low throughout the experiment. Even at the initial conditions, when H_2_ penetrated more than 5 mm (**Figure 3**), a large fraction of the manure was net-producing CO_2_, and the diffusional supply from this production must have prevented CO_2_ limitation of the process near the membrane and buffered the potential increase in pH.

### Hydrogenotrophic Community at Mesophilic Conditions: Microstructure and Control by the CO_2_ Supply

The start-up of an anaerobic reactor may require some time (weeks) but once established the populations of the different groups of fermenters, acetogens and methanogens are active and balanced (Griffin et al., 1998). As pointed by Díaz et al. (2015), the introduction of H_2_ into an anaerobic reactor does not require the use of specific strains, and it is possible to use for instance unspecific anaerobic sludge inoculum to produce an active hydrogenotrophic methanogenic reactor. The relatively slow growth rates of acetogens and acetoclastic methanogens result in relatively long reactor start-up times before a stable methane production can be obtained (Luo and Angelidaki, 2013; Ghorbanian et al., 2014; Díaz et al., 2015). However, the supply of H_2_ to an established anaerobic reactor results in the immediate activation of the hydrogenotrophic community, a fast consumption of H_2_ and an increase in CH_4_ production (Luo and Angelidaki, 2012; Wang et al., 2013; Xu et al., 2014; Hu et al., 2015). Our data confirm the predisposition of the microbial community present in a mesophilic anaerobic reactor to metabolize an external supply of H_2_ (**Figures 4** and **5**), and also provide a relevant description of the microstructure of the process.

One of the main problems previously reported by H_2_ addition to reactors is the limitation of H_2_ transfer to the reactor fluid (Luo et al., 2012; Luo and Angelidaki, 2013; Bensmann et al., 2014) by direct supply of H_2_ gas to the reactor, resulting in substantial H_2_ concentrations in the produced biogas. The use of gas permeable membranes for the H_2_ supply makes it possible to have localized high H_2_ concentrations and consumption rates (**Figures 3** and **4**) without massive ebullition of H_2_ to the headspace. Laboratory scale membrane reactors have thus been shown to produce biogas with only trace concentrations of H_2_ in the produced biogas and a high quality of up to 99% CH_4_ (Wang et al., 2013). The performance of membrane reactors is, however, limited by the amount of H_2_ that can diffuse out of the membranes, and membrane reactors must thus contain a large surface area of permeable tubing. Hollow fiber membranes may be applied (Wang et al., 2013; Díaz et al., 2015) instead of silicone tubing, but the cost of these membranes might prohibit full scale applications.

Addition of H_2_ to anaerobic reactors can result in high rates of methanogenesis, but as the H_2_ supply is not coming from a catabolic process with a parallel CO_2_ production there may be a problem with CO_2_ depletion and rise in pH. The interruption of the CO_2_ supply as shown in **Figures 4** and **5** resulted in an immediate decrease in H_2_ consumption rate and an increase in pH (**Figures 4** and **5**). Although no external CO_2_ was supplied, the small internal CO_2_ production and CO_2_/HCO_3_^-^ pool were supporting H_2_ consumption rates for a few hours, dropping to low values after 10 h. During this period, the H_2_ consumption rates decreased progressively while the CO_2_ depletion resulted in an increase of pH to values of about 9.2 (**Figure 5**), similar to the values measured in other reactors exposed to high H_2_ loadings (Wang et al., 2013; Xu et al., 2014). This value corresponds to the pK_a_ of ammonium (9.25), which is present at high concentrations in anaerobic reactors and therefore at this pH will contribute to buffering compound in the reactor. Several microbial processes may be inhibited at such high pH, resulting in the stop of the anaerobic digestion and the destabilization of the reactor, finally leading to the dramatic decrease in H_2_ consumption and CH_4_ production (Wang et al., 2013). Especially the high concentration of free NH_3_ at high pH values may be very inhibitory for both methanogens and syntrophic acetogens (Wang et al., 2015). The supply of CO_2_ together with the H_2_ through the silicone membrane following CO_2_ depletion resulted in a partial recovery of the H_2_ consumption rates (**Figure 5**). The optimal CO_2_ content in the feeding gas producing the highest H_2_ consumption rates was 5.7% (**Figures 5** and **6**), which seems to be far away for the theoretical 17.8% CO_2_ needed to react with the 71% H_2_ following a 1:4 stoichiometry for hydrogenotrophic methanogenesis. However, the CO_2_ permeability in silicone membrane is about five times higher than for H_2_ (Robb, 1968), resulting in higher fluxes through the membrane. Consequently, the CO_2_ supplied by the membrane was enough to support the high biological demand from the hydrogenotrophic methanogenesis, decreasing the pH to values below 8 (**Figures 5** and **6**). pH values below 8 are considered as optimal for anaerobic digestion (Luo and Angelidaki, 2013; Wang et al., 2013) and allow for the highest CH_4_ upgrade (Wang et al., 2013). Higher increases in the proportion of CO_2_ did not increase the H_2_ consumption rates (**Figures 5** and **6**), but decreased the pH of the matrix close to the membrane.

A balanced CO_2_ supply, being external or internal, is crucial for maintaining reactor performance by addition of H_2_ to a methanogenic reactor. The internal capacity of the reactor to produce CO_2_, including the extraction from bicarbonate resulting in moderate pH increases, should thus not be exceeded to maintain stable microbial processes. Addition of H_2_ to a methanogenic reactor through a membrane or by bubbling will probably mostly be used to upgrade the methane content of the biogas by reduction of the high CO_2_ levels present in the slurry, but it may be an advantage to have the possibility for adding more CO_2_ along with the H_2_ to facilitate recovery in reactors temporally exposed to an overdose of H_2_. The present study is from a membrane reactor, and methanogenic membrane reactors may be difficult to implement at full scale. The results illustrate, however, that H_2_ may be depleted at a very short distance from a rising H_2_ bubble as the penetration of H_2_ was ∼0.5 mm from the membrane into the digestate. Apart from a gradual rise in bulk pH, a supply of H_2_ to biogas reactors may thus only have local consequences on microbial transformations such as interspecies H_2_ transfer.

## Author Contributions

All the authors designed the study. EG-R and NR carried out the experiments. All authors interpreted the data. EG-R and NR wrote the manuscript with input from all co-authors.

## Conflict of Interest Statement

The authors declare that the research was conducted in the absence of any commercial or financial relationships that could be construed as a potential conflict of interest.

## Footnotes

1 http://www.nordpoolspot.com/historical-market-data

2 http://www.unisense.com/files/PDF/Diverse/Seawater\%20\&\%20Gases\%20table.pdf
